# Author Correction: A standard for near-scarless plasmid construction using reusable DNA parts

**DOI:** 10.1038/s41467-019-11859-6

**Published:** 2019-08-21

**Authors:** Xiaoqiang Ma, Hong Liang, Xiaoyi Cui, Yurou Liu, Hongyuan Lu, Wenbo Ning, Nga Yu Poon, Benjamin Ho, Kang Zhou

**Affiliations:** 1 Department of Chemical and Biomolecular Engineering, National University of Singapore, Singapore, 119077 Singapore; 2 Disruptive & Sustainable Technologies for Agricultural Precision, Singapore-MIT Alliance for Research and Technology, Singapore, 138602 Singapore

**Keywords:** Synthetic biology, Genetic engineering, Metabolic engineering

Correction to: *Nature Communications* 10.1038/s41467-019-11263-0, published online 23 July 2019.

The originally published version of this Article contained an error in Figs. 6 and 7. In panels 6c and 7b, the structure for E4p was incorrect. The incorrect versions of these panels appear below:

Fig. 6

Fig. 7

The correct version as Figs. 6 and 7 respectively.

Fig. 6

Fig. 7

These errors have been corrected in both the PDF and HTML versions of the Article.

