# Supplementary material for: A standard for near-scarless plasmid construction using reusable DNA parts
Source: Nat Commun. 2019 Jul 23;10:3294. doi: 10.1038/s41467-019-11263-0 (PMC6650416; doi:10.1038/s41467-019-11263-0)
Supplement: Supplementary file 1 — Supplementary Information [file 41467_2019_11263_MOESM1_ESM.pdf]

SUPPLEMENTARY INFORMATION

A standard for near-scarless plasmid construction using reusable DNA parts

Ma et al.

SUPPLEMENTARY FIGURES

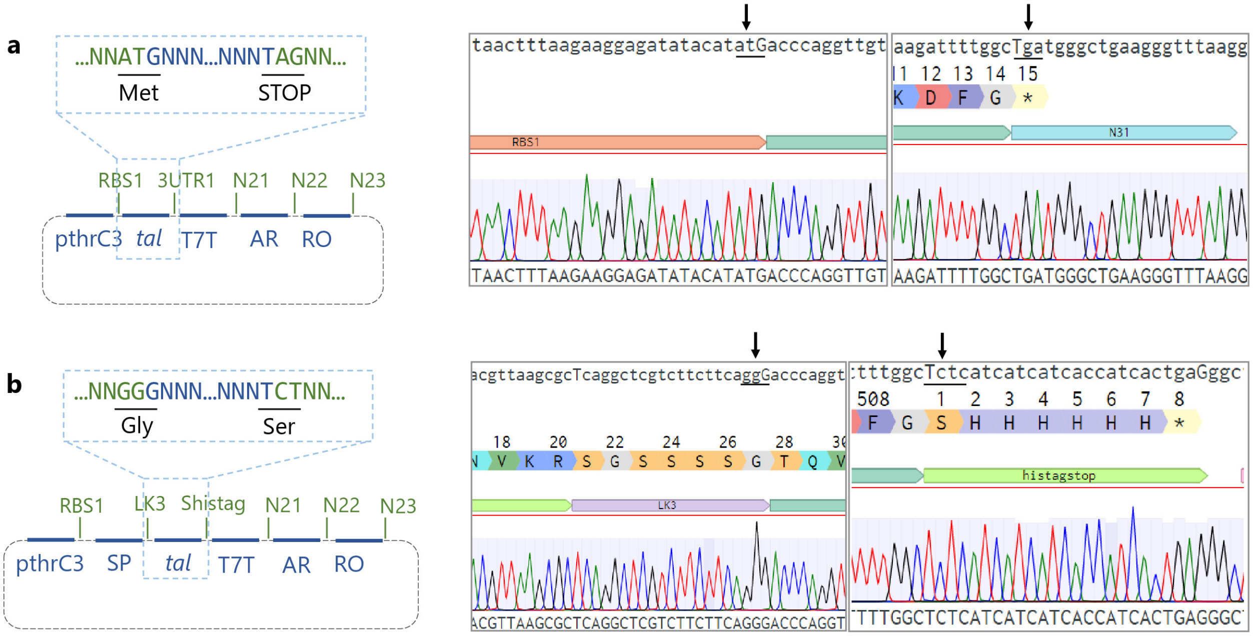

Supplementary Figure 1 GT standard (GTS) enables placing the same gene into a standalone expression cassette or into a fusion protein without leaving a scar. (**a**) *tal* (a gene encoding tyrosine ammonia-lyase) was expressed by a standalone expression cassette containing a thrC3 promoter (an auto-inducible promoter[1]) and T7 terminator (T7T). Met: start codon encoding a methionine; STOP: stop codon. Sequencing results confirmed that the desired sequences have been successfully incorporated into the plasmid at the junction regions. Alignment between sequencing data and template are as shown in the boxes on the right. The black arrows indicate the start (ATG) and stop (TGA) codons, which were correctly incorporated into the open reading frame of the gene. RBS1: ribosome binding site; Shistag: stop codon and sequence encoding a six histidine-tag (histag). N21, N22, N23: non-function barcode; AR: Antibiotic resistance marker ($Spec^R$); RO: Replication origin (pMB1); (**b**) *tal* was fused with a signal peptide[2] (SP) via a linker which consists of Glycerine (G) and Serine (S), inherently forming a fusion protein with a histag attached to its C-terminus. Sequencing results confirmed that the presence of two pre-designed sequences that encode the connecting amino acid residues within the constructed plasmid (indicated by the black arrows).

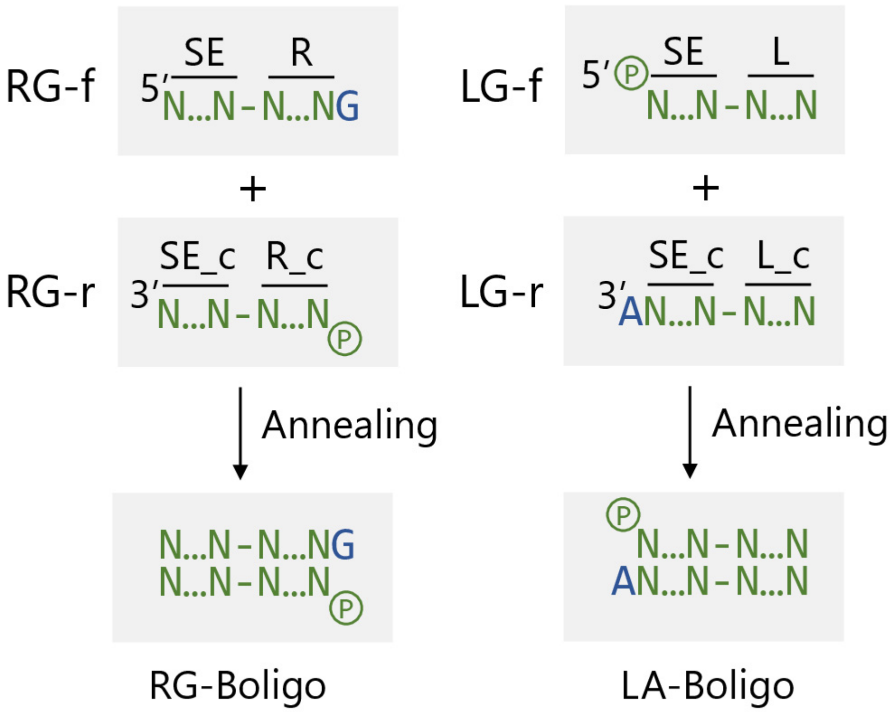

Supplementary Figure 2 To prepare conventional oligos (**Fig. 2a**), the Boligos (RG-Boligo and LA-Boligo) were annealed by using two complementary single strand oligos (RG-f/RG-r and LG-f/LG-r), one of which is shorter than the other one by one nucleotide. All oligos used to create conventional oligo are listed in **Supplementary Table 3**.

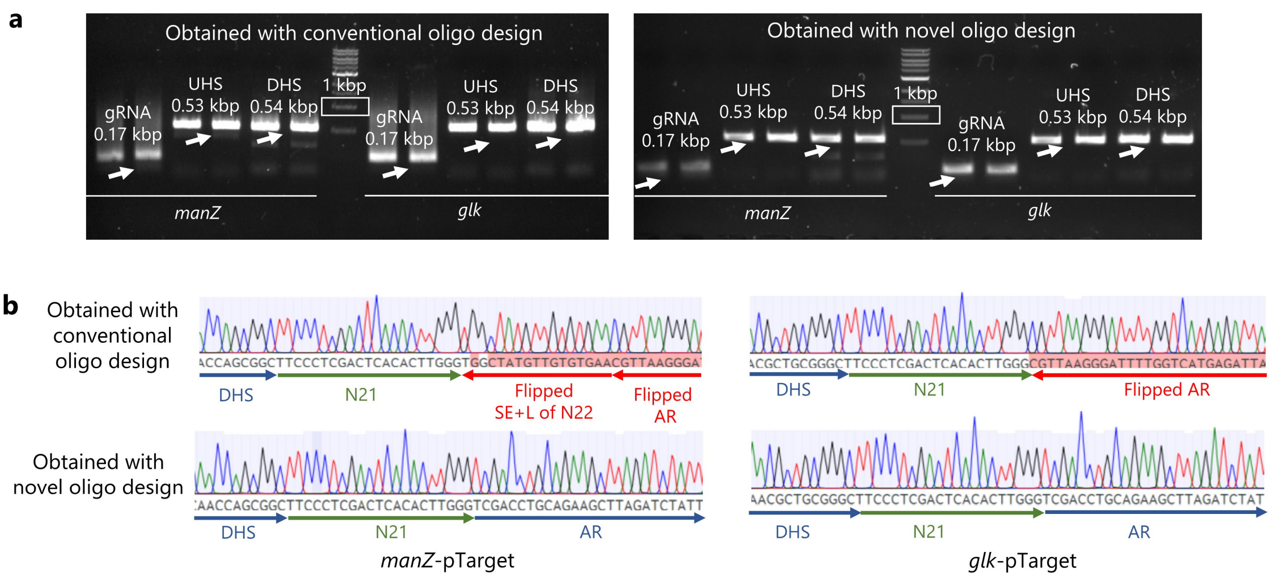

Supplementary Figure 3 (**a**) To construct the other two plasmids (*manZ*-pTarget and *glk*-pTarget) as described in **Fig. 2e**, the barcoded fragments were amplified through ligation PCR. The templates used in ligation PCR were prepared by using conventional oligo design and novel oligo design. The same three sets of Aoligo described in **Fig. 2a** and **2b** were used accordingly. The desired bands are indicated by using white arrows, and one sample was loaded into two lanes. DNA marker with 1 kbp size is highlighted in white rectangular box. Smears were observed when conventional oligo design was used. AR: Antibiotic resistance marker, gRNA: guide RNA, UHS: upstream homologous sequence, DWS: downstream homologous sequence. (**b**) Sequencing results suggested that the plasmids (*nupG*-pTarget and *glk*-pTarget) constructed by using conventional oligos design had the undesired insertion (flipped partial barcode of N22) and flipped fragment (AR was flipped). The red arrow and text indicate the problematic sequences. Sequencing results of the plasmids constructed with novel oligo design were shown to be correct.

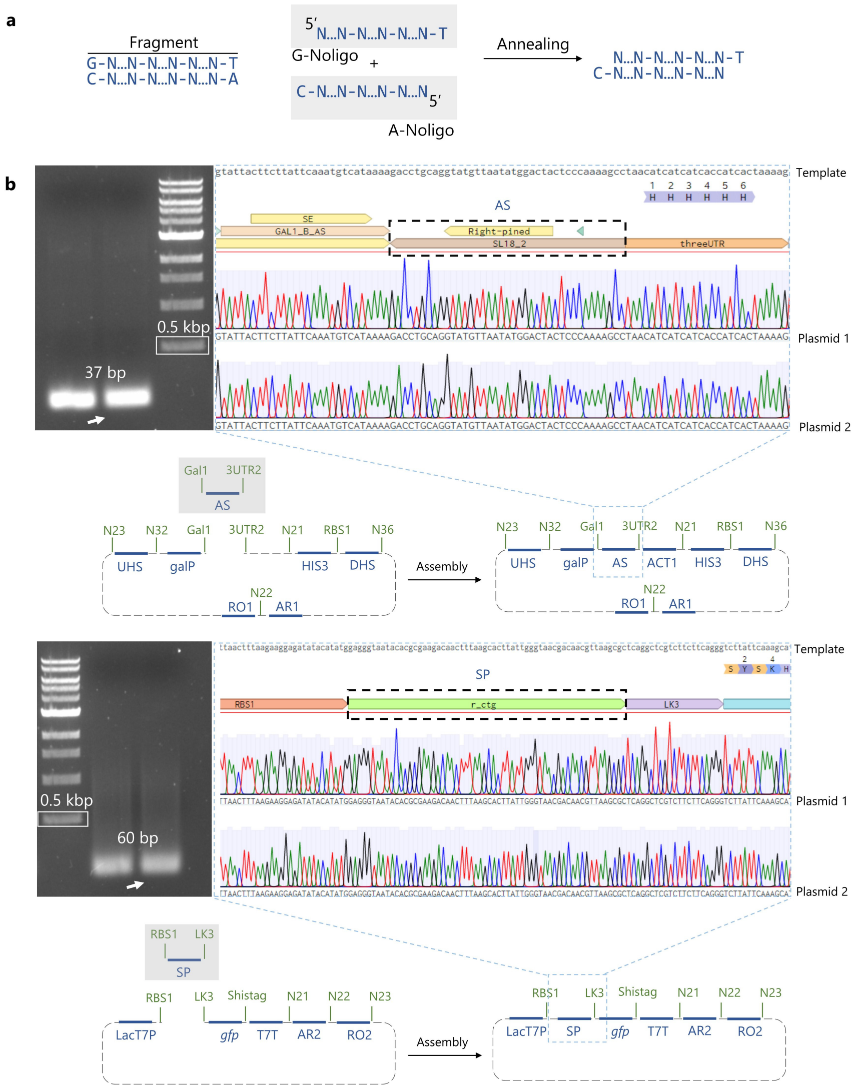

Supplementary Figure 4 Creating activated fragment by annealing Noligos. (**a**) The oligo used to create the sense strand is termed as G-Noligo (the sense strand starts with G from its 5′ end). G-Noligo is the same to the sense strand (5′ to 3′) sequence except that it does not contain the first nucleotide (G). The oligo to create the anti-sense strand is termed as A-Noligo (the antisense strand starts with ‘A’ from its 5′ end). A-Noligo is the same the antisense strand (5′ to 3′) sequence except that it does not contain the first nucleotide (A). (**b**) Two short fragments can be efficiently amplified after barcoding, and assembled into two plasmid backbones. The desired bands are indicated by using white arrows, and one sample was loaded into two lanes. DNA marker with 0.5 kbp size is highlighted in white rectangular box. For each plasmid, sequencing results confirmed that two plasmids extracted from two positive colonies had the accurate sequences at the junction region. AS (SL18-2): antisense RNA used to alter protein expression; SP (r_ctg): signal peptide used to create secretion expression system in *E. coli* as shown in **Supplementary Figure 1b**. RO1: *E. coli* replication origin (pUC); AR1: *E. coli* antibiotic resistance marker (Amp^R); UHS: Upstream homologous sequence; galP: galactose promoter from *Saccharomyces cerevisiae*; ACT1: terminator from *S. cerevisiae;* HIS3: selection marker from *S. cerevisiae*; DHS: Downstream homologous sequence; RO2: *E. coli* replication origin (pMB1); AR2: *E. coli* antibiotic resistance marker (Spec^R); LacIT7p: LacI repressor expression cassette with T7 promoter; *gfp*: a gene encoding green fluorescence protein; T7T: T7 terminator.

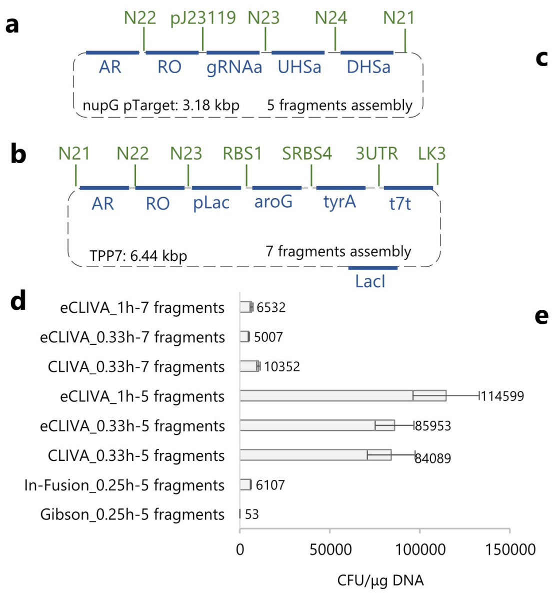

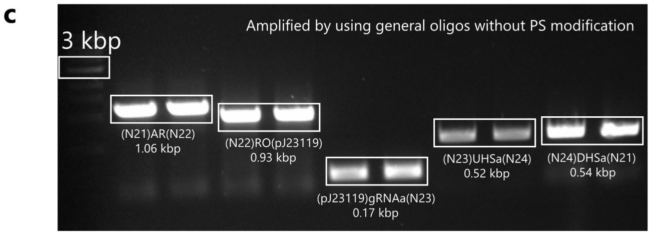

e

Assembly methods	Assembly efficiency	Sequencing accuracy	Assembly accuracy (%)
Gibson_0.25h-5 fragments	2/3	2/2	66.7
In-Fusion_0.25h-5 fragments	5/6	2/2	83.3
CLIVA_0.33h-5 fragments	6/6	2/2	100
eCLIVA_0.33h-5 fragments	6/6	2/2	100
eCLIVA_1h-5 fragments	6/6	2/2	100
CLIVA_0.33h-7 fragments	6/6	2/2	100
eCLIVA_0.33h-7 fragments	6/6	2/2	100
eCLIVA_1h-7 fragments	6/6	2/2	100

Supplementary Figure 5 Compatibility of GTS with various DNA assembly methods. Plasmids assembled by using (**a**) five- and (**b**) seven-fragment. The information of fragments and barcodes used can be found in the captions of **Fig. 2** and **Fig 6**. (**c**) Non-modified Aoligos were used to amplify the barcoded fragments that would be assembled by using Gibson method and In-fusion cloning. Five barcoded fragments were amplified from ligation products. DNA marker with 3 kbp size is highlighted in white rectangular box. The desired bands were indicated by white rectangular boxes. (**d**) The transformation efficiencies of 5- and 7-fragment assembly were evaluated by using CFU/µg DNA (three independent transformations were done by using same ligation products). CLIVA and eCLIVA obviously outperformed Gibson method and In-fusion cloning in 5-fragment assembly, and eCLIVA (eCLIVA_0.33h_5 fragments) did not work better than the original CLIVA method (CLIVA_0.33h_5 fragments) in term of assembly accuracy. Surprisingly, in 7-fragment assembly, the original CLIVA method (CLIVA_0.33h_7 fragments) worked better than eCLIVA (eCLIVA_ 0.33h_7 fragments and eCLIVA_1h_7 fragments) in term of transformation efficiency (CFU/µg DNA). (**e**) Assembly efficiency and accuracy obtained by using various methods. Assembly accuracy was product of assembly efficiency (colony PCR) and sequencing accuracy. We only took one plate for colony PCR verification as we used same ligation products in all transformations. Six colonies were randomly picked from the plate in colony PCR. In 5-framgnet assembly using Gibson method, only 3 colonies appeared on one plate, so we used all of them in colony PCR. All the sequencing results of the plasmids extracted from positive colonies showed positive.

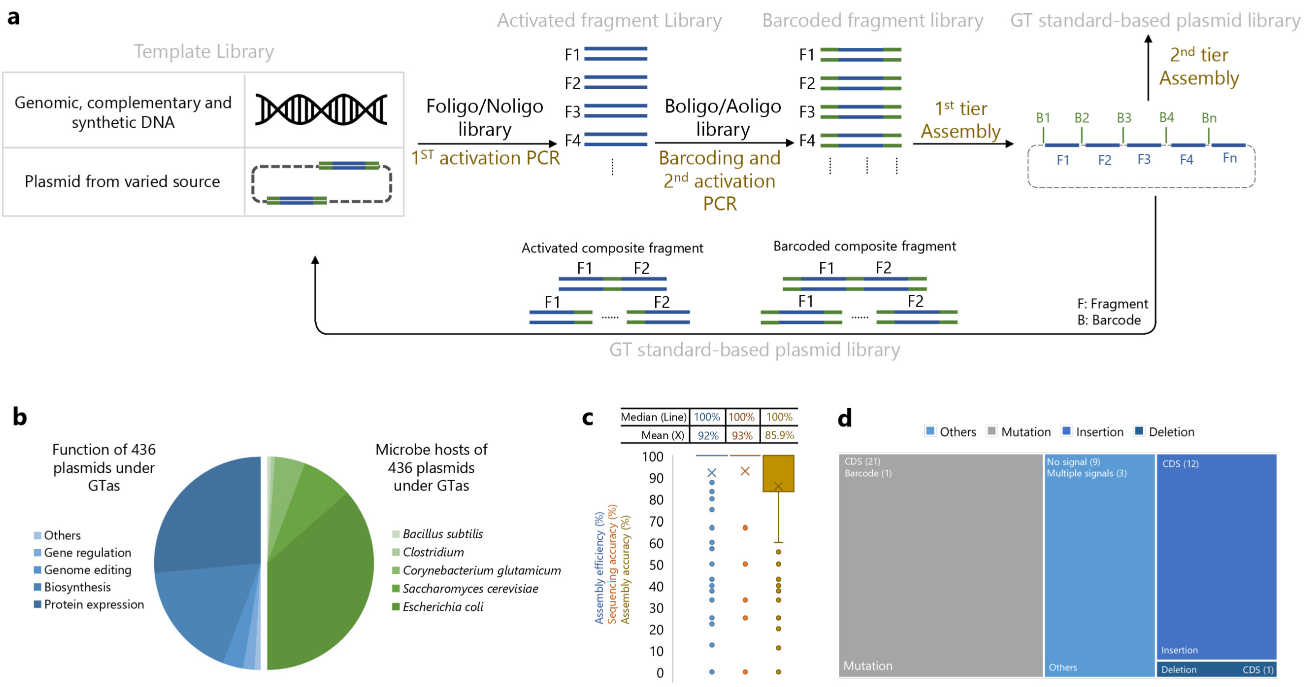

Supplementary Figure 6 GTS workflow and analysis of 436 plasmids constructed under GTS in our lab. (**a**) GTS-based library building workflow. This library contains templates, oligos and plasmids. (1) For preparation of activated fragments, Foligos or Noligos are used, and the 1st activation PCR is required if Foligos are used. (2) For preparation of barcoded fragments, Boligos are required, followed by the 2nd activation PCR which is done by using corresponding Aoligos. The barcoded fragments acquired can be assembled into plasmids in the first-tier by using Gibson, In-fusion cloning or CLIVA methods. (3) Plasmids constructed under GTS in the first-tier can be deposited into template library for extracting activated composite fragment through the 1st activation PCR by using a new pair of Foligos. Note that the internal restriction enzyme cutting site should be avoided if non-modified Foligos are used (**Fig. 4**). This obtained fragments can be barcoded by a new set of Boligos, and be activated through the 2nd round of PCR as barcoded composite fragment. With this iterative process, both flexibility of combination of activated fragments and utilization of activated fragments can be maximized, resulting in an ever-expanding GTS-based library with more versatile genetic parts. Meanwhile, the plasmids constructed in the first-tier can be also used in the second-tier for constructing more complex plasmids by using various DNA assembly methods. (**b**) Box and whisker analysis of assembly efficiency, sequencing accuracy and assembly accuracy of the 436 plasmids constructed under GTS. Assembly efficiency was evaluated by colony PCR. Assembly accuracy was product of assembly efficiency and sequencing accuracy. The outlier points that lie either below the lower whisker line or above the upper whisker line are displayed as small dots. The mean marker (X) of assembly efficiency, sequencing accuracy and assembly accuracy are presented. The medians of three data sets are presented in lines on the top of boxes. (**c**) Analysis of undesired sequencing errors of plasmid. Mutations (single and multiple bases) caused during PCR amplification were found in coding sequence (CDS) and barcode regions. Insertion (single base and multiple bases) and deletion (single base) were observed in CDS region. Scenarios of no single and multiple signals were also encountered, which were possibly due to the contamination of cell culture and plasmid extraction, poor binding efficiency of primer used in sequencing, or the instability of the constructed plasmid in *E. coli*. (**d**) Function and host analysis of the 436 plasmids constructed under GTS. All the raw data used in this figure can be found in **Supplementary Data 2**.

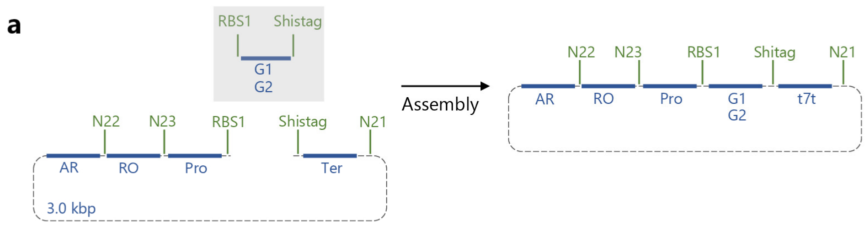

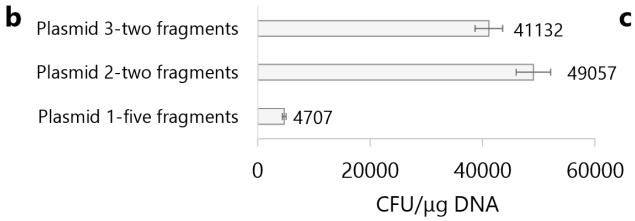

c

Plasmid and fragment number	Assembly efficiency	Sequencing accuracy	Assembly accuracy (%)
Plasmid 1-five fragments	6/6	2/2	100
Plasmid 2-two fragments	6/6	2/2	100
Plasmid 3-two fragments	5/6	2/2	83.3

Supplementary Figure 7 Type IIS RE-based barcoding method verification. (**a**) Two barcoded fragments (G1 and G2) were cloned into a pre-prepared plasmid backbone by using compatible barcode set. Blue thick horizontal bars represent fragments: G1: gene 1; G2: gene 2; t7t: T7 terminator; AR: antibiotic resistance marker ($Spec^R$); RO: replication origin (pMB1); Pro: promoter. Green texts indicate barcodes; N21, N22 and N23: non-functional connectors; RBS1: ribosome binding site; Shistag: stop codon and the sequence encoding histag. (**b**) The transformation efficiencies of assembling these three plasmids were evaluated based on CFU/μg DNA. (**c**) The assembly efficiency, sequencing accuracy and assembly accuracy of these three plasmids.

a

Barcoded fragments	Number in the right figure
(N21)SpecR(N22)	0 (Existed barcoded fragment)
(N22)pSC101(N23)	1
(N22)pAC(N23)	2
(N22)pMB1(N23)	3
(N22)pUC(N23)	4
(N23)lacIT7(RBS1)	5
(N23)pLac(RBS1)	6
(RBS1)aroG(SRBS4)	7
(RBS1)tyrA(SRBS4)	8
(SRBS4)tyrA(N31)	9
(SRBS4)aroG(N31)	10
(N31)t7t(N21)	11
(N31)t7t(LK3)	12
(LK3)LacI(N21)	13

b

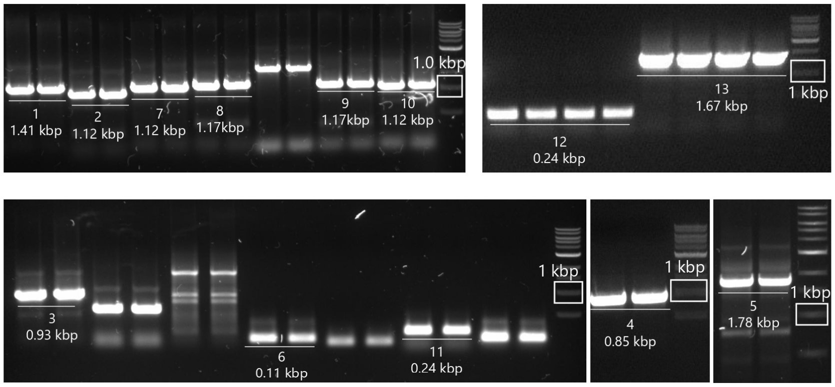

c

Plasmid name	Fragment number	Plasmid size (kbp)	Barcoded fragments used in assembly	CFU/µg DNA	Correct colony number	Colony tested number	Assembly efficiency	Correct sequenced plasmid number	Sequenced plasmid number	Accuracy of sequencing	Description of failed sequencing	Accuracy of assembly (%)
TPP1	6	6.7	0+1+5+7+9+11	4067	5	6	83	1	1	100		83
TPP2	6	6.4	0+2+5+7+9+11	4673	5	6	83	1	1	100		83
TPP3	6	6.2	0+3+5+7+9+11	9154	6	6	100	1	1	100		100
TPP4	6	6.1	0+4+5+7+9+11	5250	5	6	83	1	1	100		83
TPP5	7	6.7	0+1+5+7+9+12+13	1135	6	6	100	1	1	100		100
TPP6	7	6.4	0+2+5+7+9+12+13	3358	4	6	67	1	1	100		67
TPP7	7	6.2	0+3+5+7+9+12+13	3780	6	6	100	1	2	50	One insertion inside of CDS	50
TPP8	7	6.1	0+4+5+7+9+12+13	3969	6	6	100	1	3	33	Two mutations inside of CDS	33
TPP9	6	6.7	0+1+5+8+10+11	2105	3	6	50	1	1	100		50
TPP10	6	6.4	0+2+5+8+10+11	8912	6	6	100	1	1	100		100
TPP11	6	6.2	0+3+5+8+10+11	2254	6	6	100	1	1	100		100
TPP12	6	6.1	0+4+5+8+10+11	8318	6	6	100	1	1	100		100
TPP13	7	6.7	0+1+5+8+10+12+13	531	6	6	100	1	1	100		100
TPP14	7	6.4	0+2+5+8+10+12+13	2820	4	6	67	1	1	100		67
TPP15	7	6.2	0+3+5+8+10+12+13	3962	6	6	100	1	1	100		100
TPP16	7	6.1	0+4+5+8+10+12+13	3350	6	6	100	1	1	100		100

Supplementary Figure 8 Construction of 16 plasmids for producing tyrosine. (**a**) The list of 13 barcoded fragments used for construction of plasmids TPP1 to TPP16. The barcoded fragment [(N21)Spec^R(N22)] was prepared in the previous experiment (**Fig. 2c**), which can be directly used for plasmid construction. (**b**) Analysis of PCR products of barcoded fragments (1 to 13) demonstrated that all the 13 fragments were efficiently amplified. DNA marker with 1 kbp size of was highlighted in white rectangular box. It should be noted that the barcoded fragments were not prepared in one batch since some of barcoded fragments were used for other plasmids constructions. The white lines and number indicate the correct amplicons of barcoded fragments. (**c**) Summary of 16 plasmids construction. TPP1 to TPP4 and TP9 to TP12 were constructed by using 6 fragments, and TPP5 to TPP8 and TP13 to TP16 were constructed by using 7 fragments as the repressor fragment was needed when *Lac* promoter was used (**Fig. 6a** and **6b**). For each plasmid construction, six randomly picked colonies were tested to evaluate assembly efficiency. The sequencing of plasmids extracted from positive colonies covered the key regions of plasmid (e.g., RBS, 5′ untranslated region [5UTR], 3′ untranslated region [3UTR] and coding sequence [CDS]). Transformation efficiency of ligation product from each plasmid construction was evaluated by using CFU/µg DNA. Assembly accuracy was a product of assembly efficiency (Colony PCR) and sequencing accuracy.

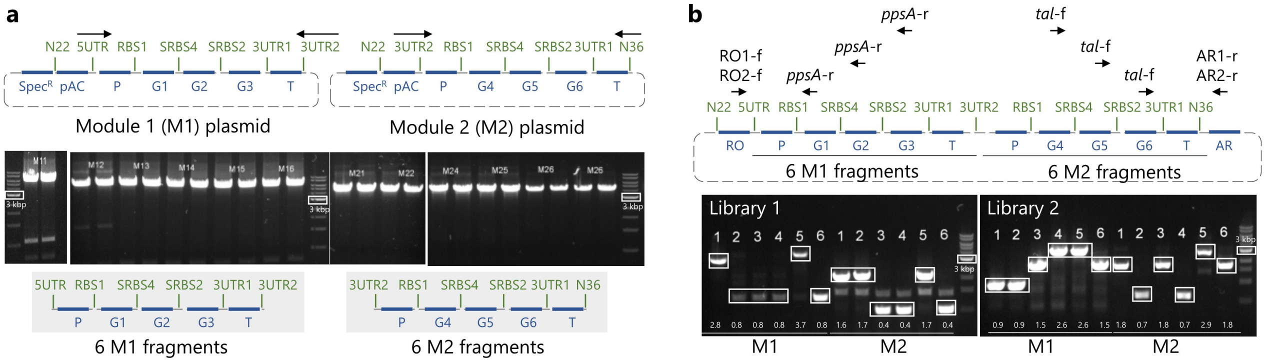

c

Gene order in operon	Library 1	Expected amplicon's length (kbp)	Library 2	Expected amplicon's length (kbp)
ppsA/aroE/tktA		0.8		0.9
ppsA/tktA/aroE		0.8		0.9
tktA/aroE/ppsA	pSC101+AmpR	3.7	pAC+SpecR	3.6
tktA/ppsA/aroE	(M1)	2.8	(M1)	2.6
aroE/ppsA/tktA		1.7		1.5
aroE/tktA/ppsA		3.7		3.6
aroG/tal/tyrA		1.6		1.8
aroG/tyrA/tal		0.4		2.9
tyrA/tal/aroG	pSC101+AmpR	1.7	pAC+SpecR	1.8
tyrA/aroG/tal	(M2)	0.4	(M2)	3.0
tal/tyrA/aroG		2.5		0.7
tal/aroG/tyrA		2.5		0.7

Supplementary Figure 9 Workflow for construction of combinatorial plasmid library. (**a**) M1 and M2 variates were amplified from M1 and M2 plasmids by using two sets of Aoligos (indicated with black arrows). DNA marker with 3 kbp size is highlighted in white rectangular box. (**b**) Six M1 fragments and six M2 fragments were combinatorically assembled with one plasmid backbone (barcoded) to create a mixture of 36 plasmids as a plasmid library. We used two plasmid backbones (pSC101+Amp^R and pAC+Spec^R) and created two plasmid libraries (Library 1 and Library 2). To confirm the existence of two modules in the plasmid from library 1, two sets of colony PCR, with the use of six colonies selected in random as template, were performed. The first colony PCR was done to detect the existence of the M1 by using oligos of RO1-f targeting on RO region and *ppsA*-r targeting on *ppsA* region, and it generated varied length of amplicons due to the fact that *ppsA* was possibly arranged to the three locations. In parallel, the second colony PCR was done specifically to detect the existence of the M2 by using oligos of AR1-f targeting on AR region and *tal*-r targeting on *tal* region. Similarly, the quality of library 2 was verified by using the same strategy. The amplification results proved that each plasmid library had the plasmids containing varied combination of M1 and M2 variants. The desired amplicon from each module of plasmid from two libraries are highlighted in white rectangular box. (**c**) The expected amplicon's length is listed accordingly for each module of plasmid from library 1 and 2.

a

Barcode	Left sequence	Sticky End (SE) sequence	Right sequence	Whole sequence of barcode (Bold: BsaI recognition site; "_": 4-bp SE used in second-tier assembly)	SE sources
5UTR-BsaIf	Ta	gcaatctaatctaagtt	ggtctcatgccG	Tagcaatctaatctaagtt**ggtctc**atgccG	5UTR1
5UTR-BsaIr	Ttgcccgagacc	gcaatctaatctaagtt	ttctagaaaaatG	Ttgccc**gagacc**gcaatctaatctaagttttctagaaaaATG	5UTR1
3UTR2-BsaIf	Taa	catcatcatcaccatca	ggtctcattggG	Taacatcatcatcaccatca**ggtctc**attggG	3UTR2
3UTR2-BsaIr	Tttggcgagacc	catcatcatcaccatca	ctaaaaG	Tttggc**gagacc**catcatcatcaccatcactaaaaG	3UTR2
N36-BsaIf	Tac	taagggcgaatgtac	ggtctcctagtG	Tactaagggcgaatgtac**ggtctc**ctagtG	N36
N36-BsaIr	Ttagtagagacc	taagggcgaatgtac	agG	Ttagta**gagacc**taagggcgaatgtacagG	N36

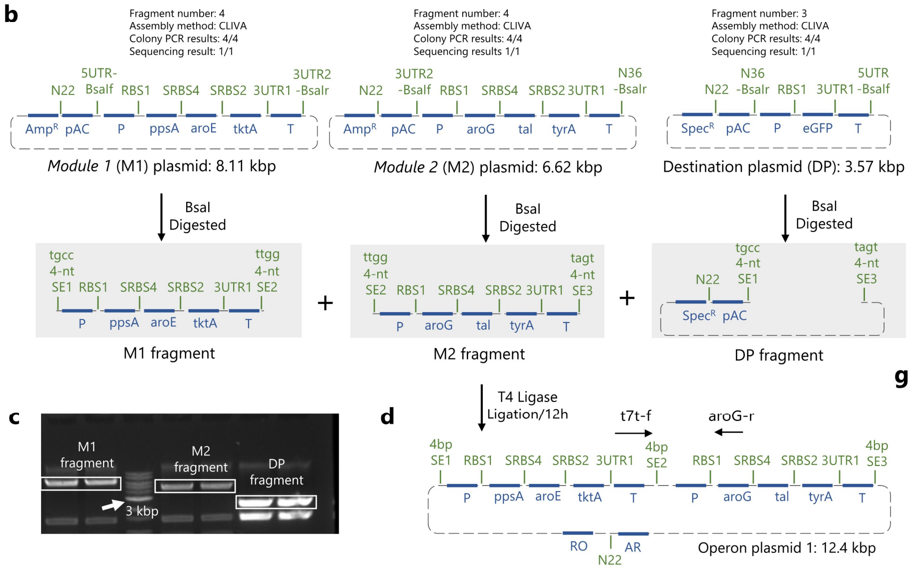

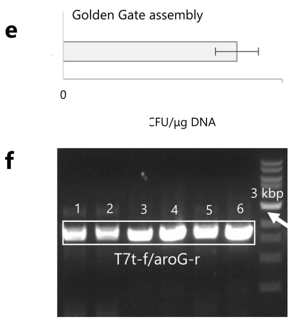

g

Assembly method in the 2nd tier	Sequencing accuracy
Golden Gate assembly	2/2

Supplementary Figure 10 Golden Gate-based two-tier workflow under GTS. (**a**) Barcodes (5UTR, 3UTR2 and N36) used in the second-tier for assembling operon M1 and M2 fragments (**Fig. 6a**) were modified to contain BsaI recognition sequence, spacer sequence, and 4-nt SE. (**b**) Three plasmids were constructed under GTS in the first-tier assembly. M1 plasmid was assembled by using 4 barcoded fragments (*ppsA*, *aroE*, *tktA* and backbone fragment [Terminator+Amp^R+pAC+Promoter]). M2 plasmid was assembled by using 4 barcoded fragments (*aroG*, *tal*, *tyrA* and backbone fragment [Terminator+AmpR+pAC+Promoter]). Destination plasmid was assembled by using 3 barcoded fragments (Spec^R, pAC and GFP expression cassette [Promoter+GFP+Terminator]). The results of colony PCR and sequencing of the three plasmids are provided on the top of plasmid schematic maps. To generate three 4-nt SEs that direct final assembly, two module plasmids and one destination plasmid were digested by using BsaI. The enzymatically digested fragments were then isolated by using gel electrophoresis, purified and assembled. The primers used for colony PCR are indicated in black arrows. (**c**) Analysis of the enzymatically digested products of three first-tier assembled plasmids. The targeted bands are highlighted in white rectangular boxes, and all three fragments were efficiently liberated from three first-tier plasmids. DNA marker with 3 kbp size is indicated by white arrow. (**e**) The transformation efficiency of three fragments assembly was evaluated by using CFU/μg DNA. (**f**) Colony PCR by using one primer targeting on M1 region and one targeting on M2 region showed that all the six randomly picked colonies were positive. The correct amplicons are highlighted in white rectangle box. DNA marker with 3 kbp size is indicated by white arrow. (**g**) Two plasmids extracted from two positive colonies were sequenced to be correct at all three 4-nt SE regions. Other regions of the final plasmid that were covered by sequencing were also correct.

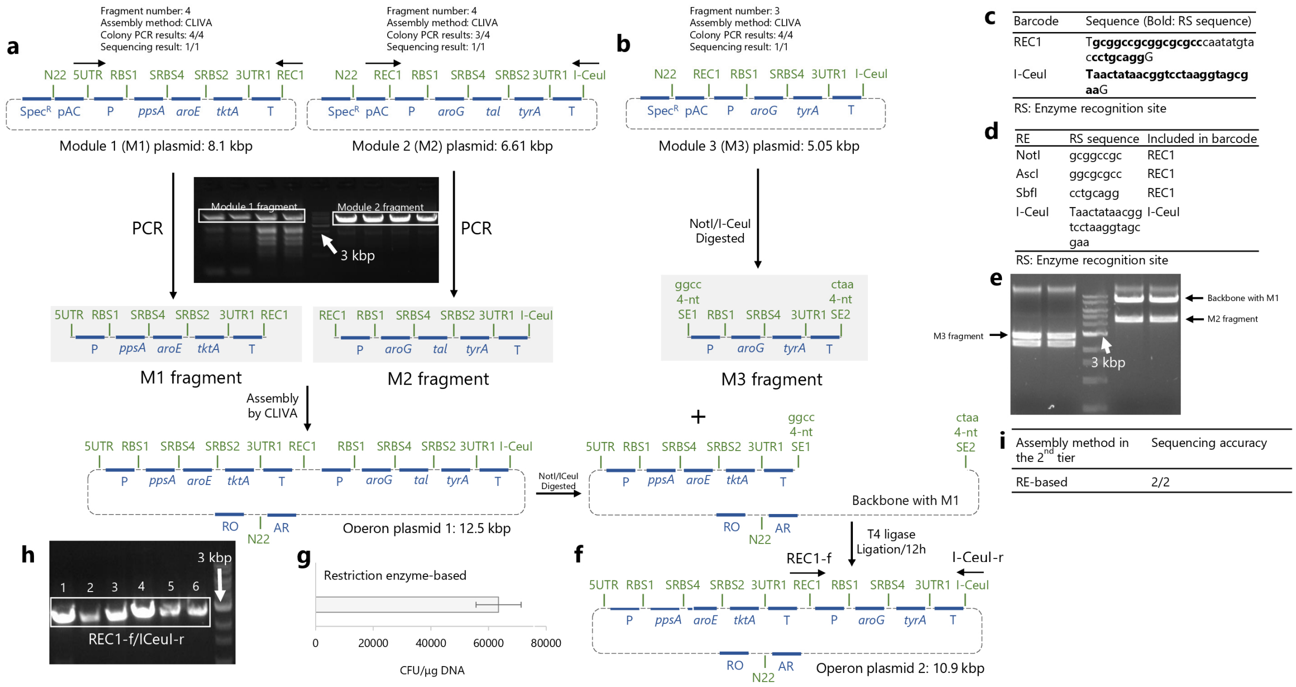

Supplementary Figure 11 RE-based two-tier workflow under GTS. (**a**) Barcodes of 5UTR, 3UTR2 and N36 used in the second-tier assembly for plasmid library construction (**Figure 5a**) were replaced by 5UTR, REC1 and I-CeuI. In the first-tier, three plasmids carrying M1, M2 and M3 were constructed by using CLIVA, and the results of colony PCR and sequencing are provided on the top of plasmid schematic maps. After that, the plasmids carrying M1 and M2 were used as template for the second round of PCR to generate M1 and M2 fragments. DNA marker with 3 kbp size is indicated by white arrow. The fragments were assembled with one plasmid backbone (RO+AR: pAC+Spec^R) to be operon plasmid 1 for pHCA production. (**b**) To convert operon plasmid 1 to be a tyrosine production plasmid, *tal* needs to be removed. M3 plasmid was constructed in the first-tier accordingly. (**c**) The sequences of REC1 barcode (containing three 8-bp RE recognition sites) and I-CeuI barcode (containing a long 19-bp RE recognition site). (**d**) The RE recognition sites included in REC1 barcode and I-CeuI barcode. (**e**) After enzymatic digestion, the backbone (containing M1) and M3 fragment were obtained. The desired bands are indicated by black arrows in gel image. DNA marker with 3 kbp size is indicated by white arrow. The purified M3 fragment was ligated with backbone containing M1 by using T4 ligase. (**f**) The operon plasmid 2 was constructed by assembling M3 with backbone carrying M1. (**g**) The transformation efficiency of two-fragment ligation was evaluated by using CFU/μg DNA. (**h**) Colony PCR by using the oligos targeting on the whole region of M3 region indicated that all six randomly picked colonies was positive. DNA marker with 3 kbp size is indicated by white arrow. (**h**) Two plasmids extracted from two positive colonies were sequenced to be correct at the regions of three barcode (5UTR, REC1 and I-CeuI). Other regions in final plasmid that were covered in sequencing are free of undesired sequence errors.

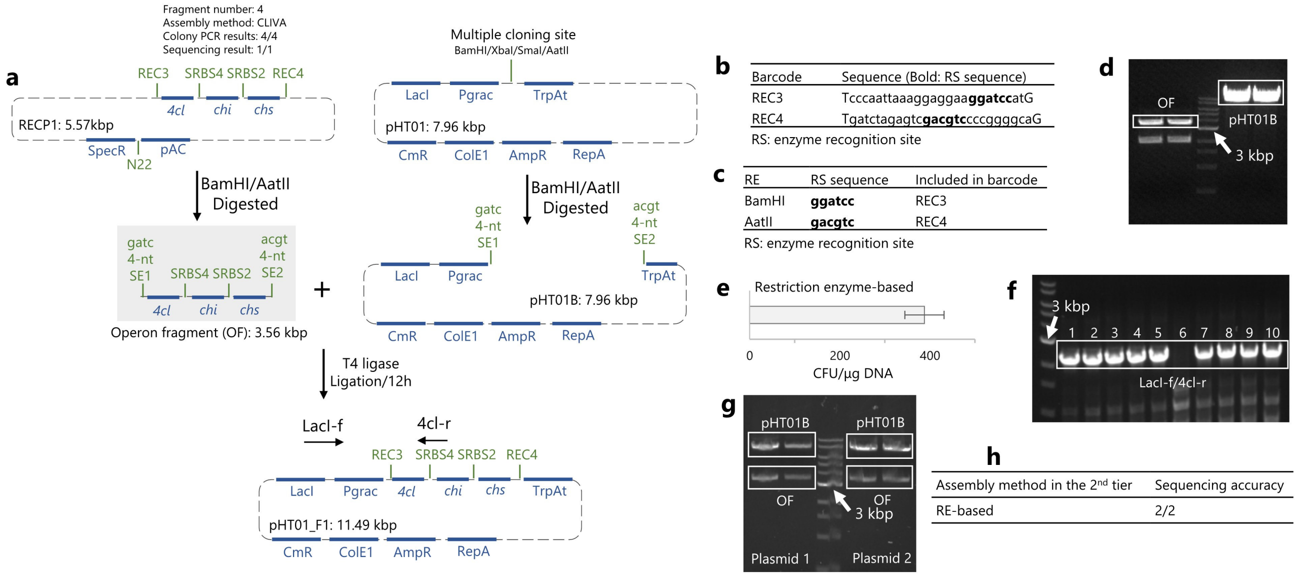

Supplementary Figure 12 Inserting an operon constructed under GTS into a given plasmid. A two-tier workflow was established for inserting a three-gene operon into pHT01, a plasmid that can be used for expressing proteins in *Bacillus subtilis*. (**a**) In the first-tier, the plasmid (RECP1) was constructed by assembling four fragments (*4cl*, *chi*, *chs* and backbone [Spec^{R}+pAC]), and the results of colony PCR and sequencing are provided on the top of plasmid schematic maps. The RECP1 and pHT01 were enzymatically digested by two REs (BamHI/AatII). The liberated operon fragment was ligated with the pHT01B fragment through 4-bp SEs by using T4 ligase. *4cl*: 4-coumaroyl-CoA ligase; *chi*: chalcone isomerase; *chs*: chalcone synthase; SpecR: antibiotic resistance marker; pAC: p15 replication origin; LacI: Lac promoter repressor expression cassette; Pgrac: grac promoter used in *B. subtilis*; TrpAt: terminator; CmR: antibiotic resistance marker used in *B. subtilis*; ColE1: replication origin used in *E. coli*; Amp^{R}: antibiotic resistance marker used in *E. coli*; RepA: replication origin used in *B. subtilis*. (**b**) The sequence of two barcodes (REC3 and REC4). (**c**) RE recognition sites included in REC3 barcode and REC4 barcode. (**d**) Gel electrophoresis analysis of enzymatically digested products of RECP1 and pHT01, and the targeted bands are highlighted in white rectangle boxes. DNA marker with 3 kbp size is indicated by white arrow. (**e**) The transformation efficiency of two-fragment ligation was evaluated by using CFU/µg DNA. (**f**) Colony PCR by using one primer (LacI-f) targeting on backbone region and one targeting on the operon region (4cl-r) indicated that 9 out of 10 randomly picked colonies was positive. DNA marker with 3 kbp size is indicated by white arrow. (**g**) RE-digestion confirmed two plasmids extracted from two positive colonies have correct operon fragment. DNA marker with 3 kbp size is indicated by white arrow. (**h**) Sequencing of these two plasmid confirmed that all the barcode regions were accurate. The other regions of the final plasmid that could be covered in sequencing were also free of sequencing errors.

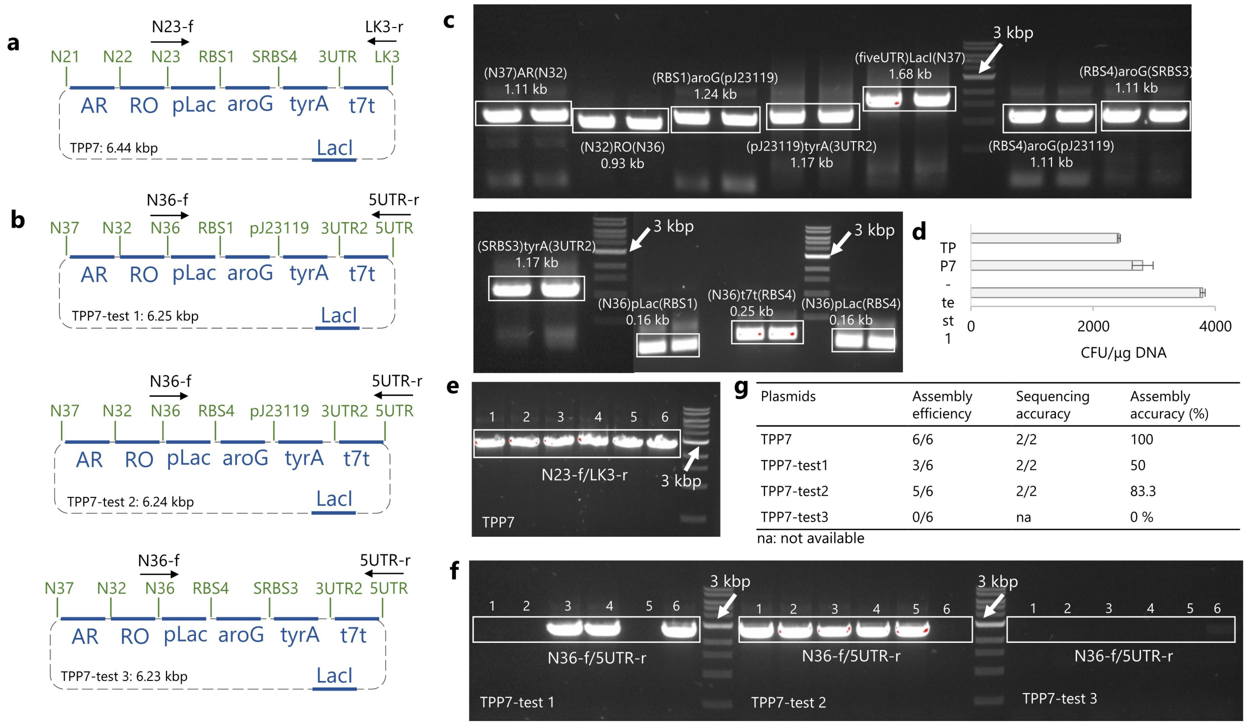

Plasmids	Assembly efficiency	Sequencing accuracy	Assembly accuracy (%)
TPP7	6/6	2/2	100
TPP7-test1	3/6	2/2	50
TPP7-test2	5/6	2/2	83.3
TPP7-test3	0/6	na	0 %

na: not available

Supplementary Figure 13 Testing various sets of barcodes in 7-fragment assembly. (**a**) The barcode set used to construct the plasmid TPP7 for overproducing tyrosine. (**b**) Six barcodes in the plasmid TPP7 were replaced in the plasmid TPP7-test1 except RBS1, and seven barcodes in the plasmid TPP7 were replaced in the plasmid TPP7-test2 and TPP7-test3. Blue thick horizontal bars represent fragments; AR: Antibiotic resistance marker (Spec^{R}); RO: Replication origin (pMB1); pLac: Lac promoter; *aroG*: a gene encoding mutated *E. coli* 3-deoxy-7-phosphoheptulonate synthase; tyrA: a gene encoding mutated *E. coli* fused chorismate mutase/prephenate dehydrogenase; t7t: T7 terminator; LacI: Lac promoter repressor expression cassette. Green texts indicate barcode; N21, N22, N23, N24, LK3, N32, N36, N37, 5UTR: non-functional connectors; RBS4: ribosomal binding site; pJ23119: a constitutive promoter; 3UTR2: 3′ untranslated region. (**c**) Analysis of PCR products of the barcoded fragments that would be assembled to be the plasmids TPP7-test1, TPP7-test2 and TPP7-test3. The desired bands were highlighted in white rectangular boxes. DNA marker with 3 kbp size is indicated by white arrow. (**d**) Transformation efficiencies of the three plasmids assembly. The PCR products of the barcoded fragments used to construct the plasmid TPP7 are shown in **Supplementary Figure 8b**. The results of colony PCR used to verify the assembly efficiency of TPP7 (**e**), TPP7-test1, TPP7-test2 and TPP7-test3 (**f**). The correct amplicons were highlighted in white rectangle boxes. (**g**) Sequencing results of the plasmid extracted from the colonies verified by colony PCR. The results indicate that all the sequenced plasmids are correct. The sequencing was performed to cover the important regions (e.g., barcode region and CDS).

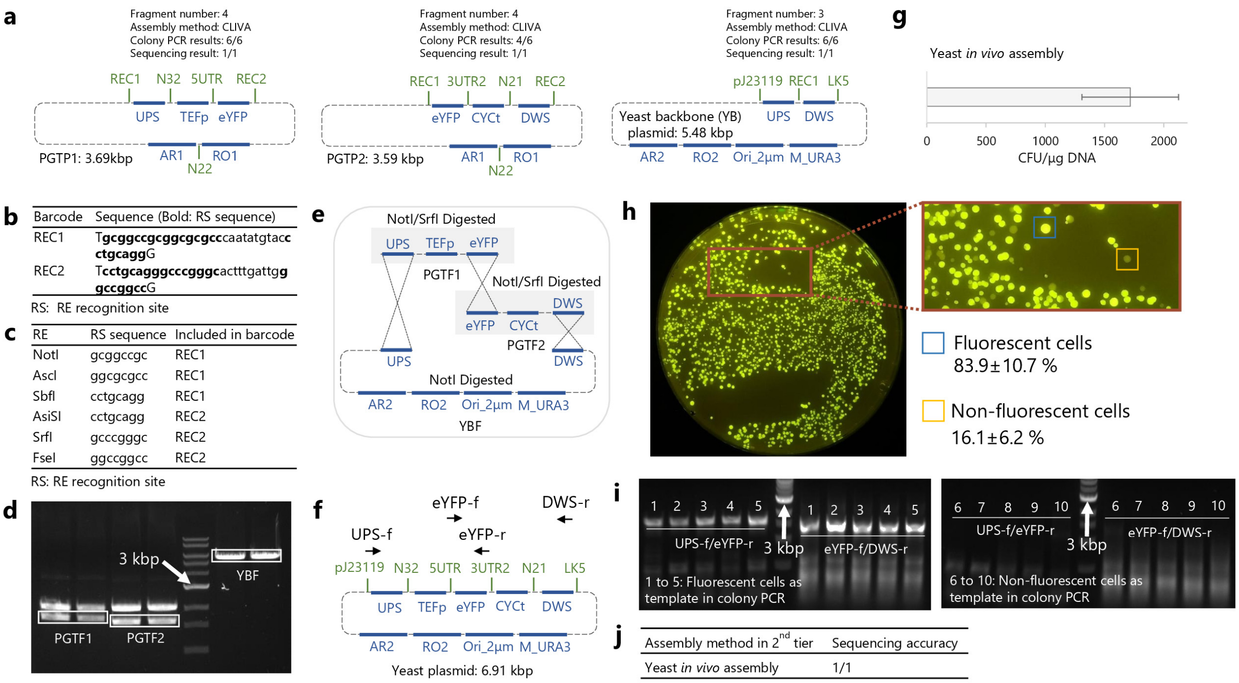

Supplementary Figure 14 Yeast *in vivo* two-tier workflow under GTS. (**a**) The first-tier assembly was performed to create three plasmids that were used to liberate the fragments for yeast *in vivo* assembly by using REs. PGTP1 was assembled by using 4 barcoded fragments (upstream sequence [UPS], TEF promoter, enhanced yellow fluorescent protein [eYFP] and backbone [AR1+RO1]). PGTP2 was assembled by using 4 barcoded fragments (eYFP, CYC terminator [CYCt], downstream sequence [DWS] and backbone [AR1+RO1]). Yeast backbone (YB) plasmid was constructed by using 3 barcoded fragments (UPS, DWS and Yeast backbone). The results of colony PCR and sequencing are provided on the top of plasmid schematic maps. RO1: *E. coli* replication origin (pMB1); AR1: *E. coli* antibiotic resistance marker (Spec^R); RO2: *E. coli* replication origin (pUC); AR2: *E. coli* antibiotic resistance marker (Amp^R). (**b**) The sequences of barcodes (REC1 and REC2), which include three 8-bp RE recognition sites on each barcode. (**c**) RE recognition sites included in REC1 barcode and REC2 barcode. (**d**) The gel electrophoresis analysis of the enzymatically digested products of three first-tier plasmids. The target amplicons are highlighted in white rectangular boxes. DNA marker with 3 kbp size was indicated by white arrow. The isolated fragments (PGTPF1, PGTP2 and YB fragment [YBF]) were purified for further assembly. (**e**) Yeast *in vivo* assembly of 3 fragments. The fragments of UPS, eYFP and DWS were served as homologous arms for guiding the recombination in *S. cerevisiae*. (**f**) The yeast plasmid constructed by assembling three fragments. The primers used in yeast colony PCR are indicated by using black arrows. (**g**) The transformation efficiency of three fragments assembly in yeast was evaluated by using CFU/μg DNA. (**h**) The colonies raised on the plate were exposed under Dark Reader in order to count the number of fluorescent cell and non-fluorescent cell. The fluorescent cell (highlighted in small blue box) and non-fluorescent cell (highlighted in small orange box) were shown in right-side zoom-in figure (highlighted in dark red box). Based on the counting of colonies raised on three plates, the percentage of fluorescent cell and non-fluorescent cell were provided. (**i**) Colony PCR confirmed that all five colonies having fluorescence used as template have correct amplicons (1.67 kbp, indicated by white lines), while no correct bands were able to be amplified from the colonies without fluorescence. DNA marker with 3 kbp size is indicated by white arrow. (**j**) One plasmid extracted from a positive yeast colony was sequenced, and the results showed that all the regions covered in sequencing were correct.

a

Number	Plasmid to be constructed (barcode used are listed in bracket between two fragments)	Barcode 1 oligos	Barcode 2 oligos	Barcode 3 oligos	Barcode 4 oligos	Barcode 5 oligos	Barcode 6 oligos	Barcode 7 oligos
P3	(N21)aadA(N22)pmb1(N23)lacIT7(RBS1)chi(histagstop)t7t	RBS1-G	Shistag-A	X	X	X	X	X
P4	(N21)aadA(N22)pmb1(N23)lacIT7(RBS1)chs(histagstop)t7t	RBS1-G	Shistag-A	X	X	X	X	X
P418	(N21)aadA(N22)p5(N23)lacIT7(RBS1)aroG mutant(RBS4stop)tyrA mutant(N31)t7t-TPP1	X	N22-G	N23-G/A	RBS1-G/A	SRBS4-G/A	3UTR1-A	X
P419	(N21)aadA(N22)p15(N23)lacIT7(RBS1)aroG mutant(RBS4stop)tyrA mutant(N31)t7t-TPP2	X	N22-G	N23-A	X	X	X	X
P420	(N21)aadA(N22)pMB1(N23)lacIT7(RBS1)aroG mutant(RBS4stop)tyrA mutant(N31)t7t-TPP3	X	N22-G	N23-A	RBS1-G	SRBS4-G/A	3UTR1-A	X
P421	(N21)aadA(N22)pUC(N23)lacIT7(RBS1)aroG mutant(RBS4stop)tyrA mutant(N31)t7t-TPP4	X	N22-G	N23-A	X	X	X	X
P422	(N21)aadA(N22)p5(N23)lacIT7(RBS1)tyrA mutant(RBS4stop)aroG mutant(N31)t7t-TPP9	X	X	X	X	X	X	X
P423	(N21)aadA(N22)p15(N23)lacIT7(RBS1)tyrA mutant(RBS4stop)aroG mutant(N31)t7t-TPP10	X	X	X	X	X	X	X
P424	(N21)aadA(N22)pMB1(N23)lacIT7(RBS1)tyrA mutant(RBS4stop)aroG mutant(N31)t7t-TPP11	X	X	X	X	X	X	X
P425	(N21)aadA(N22)pUC(N23)lacIT7(RBS1)tyrA mutant(RBS4stop)aroG mutant(N31)t7t-TPP12	X	X	X	X	X	X	X
P426	(N21)aadA(N22)p5(N23)pLac(RBS1)aroG mutant(RBS4stop)tyrA mutant(N31)t7t(LK3)LacI-TPP5	N21-G	X	N23-G	RBS1-A	X	3UTR1-G	LK3-G/A
P427	(N21)aadA(N22)p15(N23)pLac(RBS1)aroG mutant(RBS4stop)tyrA mutant(N31)t7t(LK3)LacI-TPP6	X	X	X	X	X	X	X
P428	(N21)aadA(N22)pMB1(N23)pLac(RBS1)aroG mutant(RBS4stop)tyrA mutant(N31)t7t(LK3)LacI-TPP7	X	X	X	X	X	X	X
P429	(N21)aadA(N22)pUC(N23)pLac(RBS1)aroG mutant(RBS4stop)tyrA mutant(N31)t7t(LK3)LacI-TPP8	X	X	X	X	X	X	X
P430	(N21)aadA(N22)p5(N23)pLac(RBS1)tyrA mutant(RBS4stop)aroG mutant(N31)t7t(LK3)LacI-TPP13	X	X	X	X	X	X	X
P431	(N21)aadA(N22)p15(N23)pLac(RBS1)tyrA mutant(RBS4stop)aroG mutant(N31)t7t(LK3)LacI-TPP14	X	X	X	X	X	X	X
P432	(N21)aadA(N22)pMB1(N23)pLac(RBS1)tyrA mutant(RBS4stop)aroG mutant(N31)t7t(LK3)LacI-TPP15	X	X	X	X	X	X	X
P433	(N21)aadA(N22)pUC(N23)pLac(RBS1)tyrA mutant(RBS4stop)aroG mutant(N31)t7t(LK3)LacI-TPP16	X	X	X	X	X	X	X

X: no barcode-associated oligos were used.

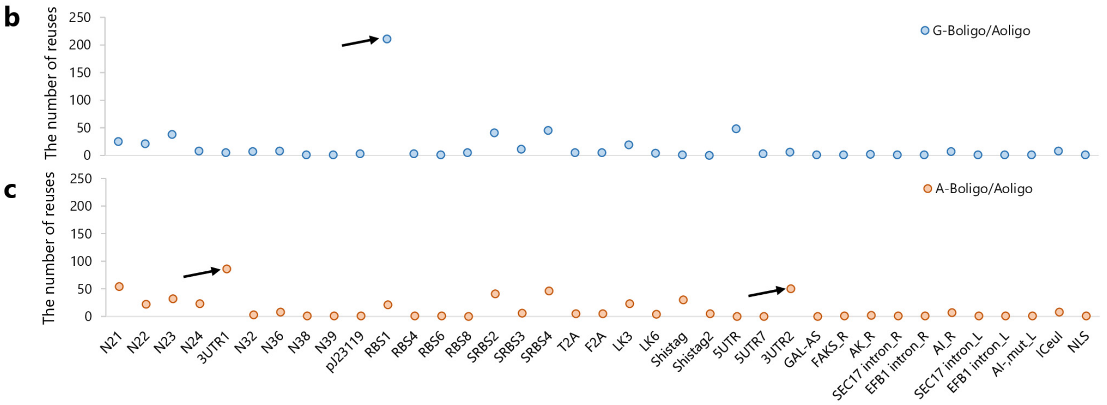

Supplementary Figure 15 The reusability of barcode-associated Aoligos and Boligos. (**a**) Examples of reusing barcode-associated oligos to prepare the barcoded fragments. For construction of P3 and P4, the G-Boligo/Aoligo of RBS1 and the A-Boligo/Aoligo of Shitag were considered to be reused as they were used for barcoding two different fragments. For construction of P418 and P419, the barcoded fragment [(N21)Spec^R(N22)] has been prepared as mentioned in **Fig. 2d**, thus no barcode-associated oligos were reused. In addition, once four barcoded fragments of RO (pSC101[p5], pAC[p15], pMB1 and pUC) were prepared, they can be reused for constructing TPP1 to TPP16. In such case, no barcode-associated oligos were needed, unless we need to change the barcoded fragment (LacIT7 was replaced by pLac barcoded with same set of barcode, and half of the barcode-associated oligos were required [G-Boligo/Aoligo of N23 and A-Boligo/Aoligo of RBS1]). In short, the reuse of the barcoded associated oligos depends on the fragment to be barcoded instead of the frequency of barcoded fragment used in various plasmid construction. Whole profile analysis of the reuse of (**b**) G-Boligo/Aoligo and (**c**) A-Boligo/Aoligo in 436 plasmids we constructed under GTS in the lab. Three barcode-associated oligos indicated by black arrows have been reused over 50 times. The averaged reuse of G-Boligo/Aoligo and A-Boligo/Aoligo is ~14 times.

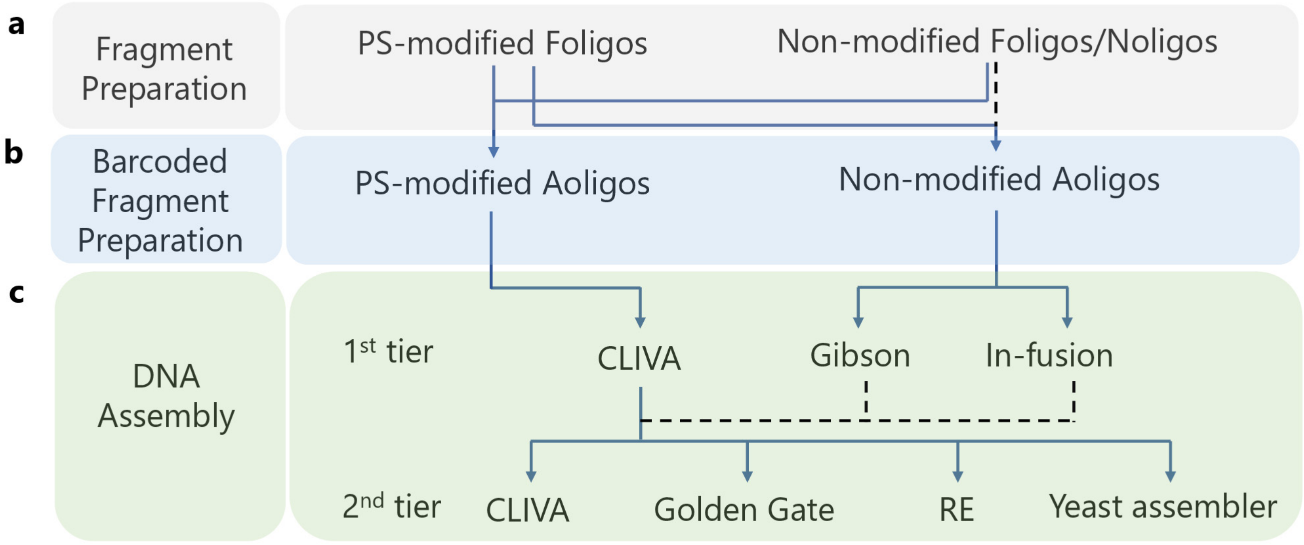

Supplementary Figure 16 The architecture of GTS workflow. (**a**) Based on barcoding method used, fragments can be amplified by using Foligos with or without PS bonds, or prepared by annealing Noligos without PS bonds. (**b**) Based on DNA assembly method used, barcoded fragments can be amplified by using PS-modified or non-modified Aoligos. (**c**) Two-tier DNA assembly workflows under GTS. The two-tier workflows from ***preparation/barcoding of fragment*** to ***assembly of barcoded fragment*** as indicated by blue solid lines have been experimentally demonstrated in this study (CLIVA: **Fig. 6a**; Golden Gate: **Supplementary Figure 10**; RE: **Supplementary Figure 11** and **Supplementary Figure 12**; Yeast assembler: **Supplementary Figure 14**). In theory, the pathways highlighted by using black dotted lines should also be applicable under GTS.

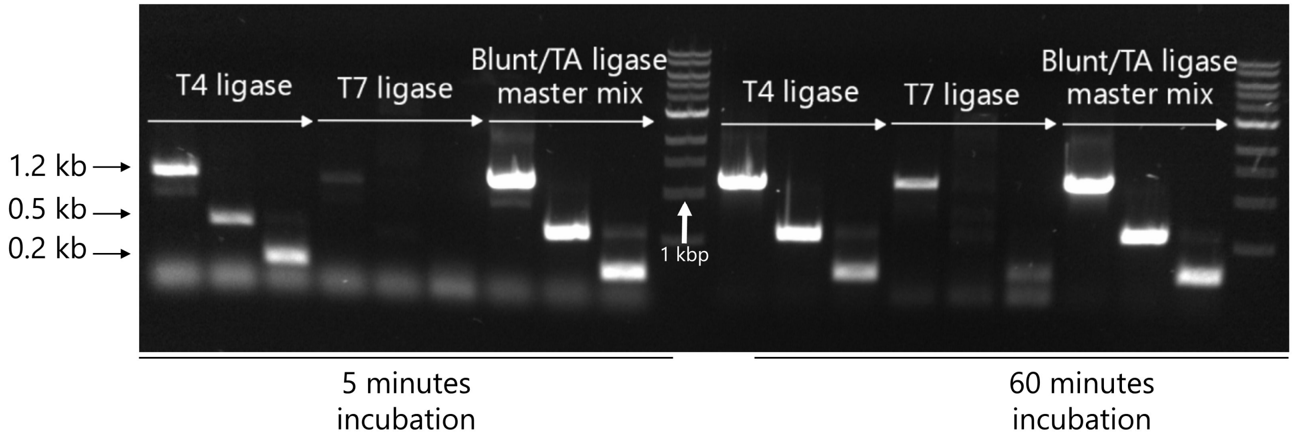

Supplementary Figure 17 Optimization of barcoding reaction by using three ligation kits. Three microliters of three fragments with various length were ligated with 0.3 µL of RG-Boligo (5UTR) and 0.3 µL of LA-Boligo (3UTR2). The ligation of Boligos with the activated fragments (containing two 1-nt SEs) was performed by using three ligation kits (T4 ligase [M0202L], T7 ligase [M0318L] and Blunt/TA ligase master mix [M0367L] from NEB). Three sets of ligation mixtures were incubated at 25 °C for 5 or 60 min. One microliter of each ligation product was used as template in PCR by using oligos of RG-Aoligo (5UTR) and LA-Aoligo (3UTR2). We found that, after 5 min incubation, Blunt/TA ligase master mix evidently outperformed the other two ligation kits in term of intensity of desired bands on the gel image. T4 ligase-mediated barcoding achieved similar results, as compared to Blunt/TA ligase master mix after 60 min of incubation. However, T7 ligase cannot efficiently barcode two shorter fragments (0.5 and 0.2 kb), since no distinct bands were observed even after incubation for 60 min. DNA marker with 1 kbp size is indicated by white arrow.

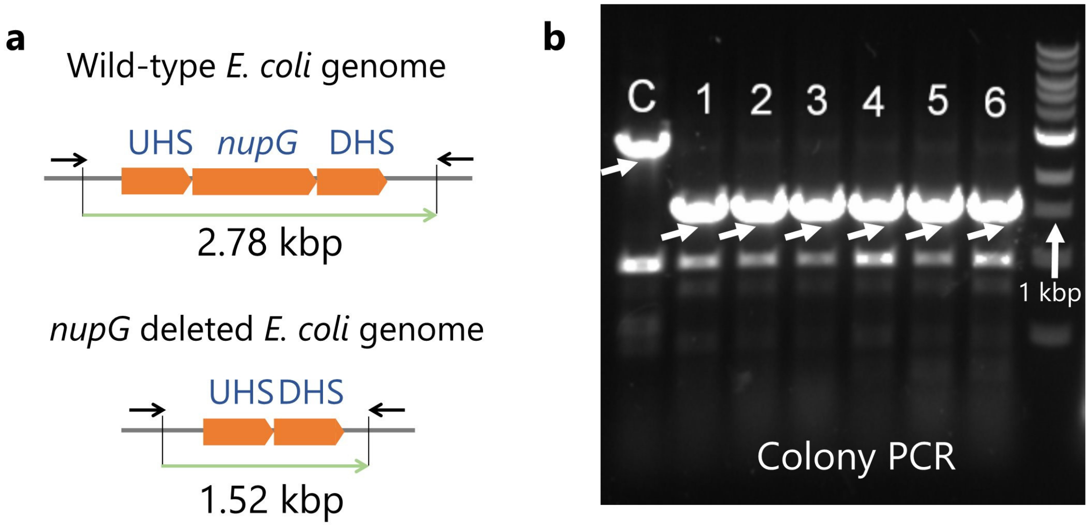

Supplementary Figure 18 Functionality test[3] of *nupG*-pTarget constructed by using novel oligo design (**Fig. 2e**). (**a**) The schematic diagram of the wild-type and the deleted *E. coli nupG locus*. Black arrows indicate the oligos used for colony PCR verification. (**b**) Colony PCR demonstrated that six randomly picked colonies had the desired deletion. The negative control (C) was performed by using wild-type *E. coli* cell as colony PCR template. White arrows indicate the desired bands. DNA marker with 1 kbp size is also indicated by white arrow at the right-side of gel image. The oligos used for colony PCR test are listed in **Supplementary Table 5**.

SUPPLEMENTARY DATA

Supplementary data are provided in separate excel files.

Supplementary Data 1
List of fragments, Foligos and Noligos used in this study

Supplementary Data 2
GTS barcode library

Supplementary Data 3
List of oligos used to prepare Boligos in this study

Supplementary Data 4
List of Aoligos used in this study

Supplementary Data 5
List of gene deletion efficiency by using pTarget plasmid constructed under GTS

Supplementary Data 6
List of oligos used in colony PCR for constructing plasmid library in this study

Supplementary Data 7
List of oligos used in colony PCR for testing various DNA assembly methods

Supplementary Data 8
List of barcoded fragments used in this study

Supplementary Data 9
Statistical analysis of construction of 436 plasmids under GTS. The raw data used for statistical analysis (**Figure 3** and **Supplementary Figure 6**) of construction of the 436 plasmids under GTS are provided in a separate excel file.

Supplementary Data 10
List of plasmids and strains constructed in this study

REFERENCE

1. Anilionyte, O., Liang, H., Ma, X., Yang, L. & Zhou, K. Short, auto-inducible promoters for well-controlled protein expression in *Escherichia coli*. *Appl. Microbiol. Biotechnol.* **102**, 7007-7015 (2018).
2. Gao, D., Wang, S., Li, H., Yu, H. & Qi, Q. Identification of a heterologous cellulase and its N-terminus that can guide recombinant proteins out of *Escherichia coli*. *Microb. Cell. Fact.* **14**, 49 (2015).
3. Jiang, Y. et al. Multigene editing in the *Escherichia coli* genome via the CRISPR-Cas9 system. *Appl. Environ. Microbiol.* **81**, 2506-2514 (2015).
