# Supplementary material for: A standard for near-scarless plasmid construction using reusable DNA parts
Source: Nat Commun. 2019 Jul 23;10:3294. doi: 10.1038/s41467-019-11263-0 (PMC6650416; doi:10.1038/s41467-019-11263-0)
Supplement: Supplementary file 13 — Description of Additional Supplementary Files [file 41467_2019_11263_MOESM13_ESM.docx]

**Title:** Supplementary Data 1 (Supplementary Data 1.xlsx)

**Description:** List of fragments, Foligos and Noligos used in this study

**Title:** Supplementary Data 2 (Supplementary Data 2.xlsx)

**Description:** GTS barcode library

**Title:** Supplementary Data 3 (Supplementary Data 3.xlsx)

**Description:** List of oligos used to prepare Boligos in this study

**Title:** Supplementary Data 4 (Supplementary Data 4.xlsx)

**Description:** List of Aoligos used in this study

**Title:** Supplementary Data 5 (Supplementary Data 5.xlsx)

**Description:** List of gene deletion efficiency by using pTarget plasmid constructed under GTS~

**Title:** Supplementary Data 6 (Supplementary Data 6.xlsx)

**Description:** List of oligos used in colony PCR for constructing plasmid library in this study

**Title:** Supplementary Data 7 (Supplementary Data 7.xlsx)

**Description:** List of oligos used in colony PCR for testing various DNA assembly methods

**Title:** Supplementary Data 8 (Supplementary Data 8.xlsx)

**Description:** List of barcoded fragments used in this study

**Title:** Supplementary Data 9 (Supplementary Data 9.xlsx)

**Description:** Statistical analysis of construction of 436 plasmids under GTS

**Title:** Supplementary Data 10 (Supplementary Data 10.xlsx)

**Description:** List of plasmids and strains constructed in this study
