# Supplementary figures and images for: A standard for near-scarless plasmid construction using reusable DNA parts
Source: Nat Commun. 2019 Jul 23;10:3294. doi: 10.1038/s41467-019-11263-0 (PMC6650416; doi:10.1038/s41467-019-11263-0)

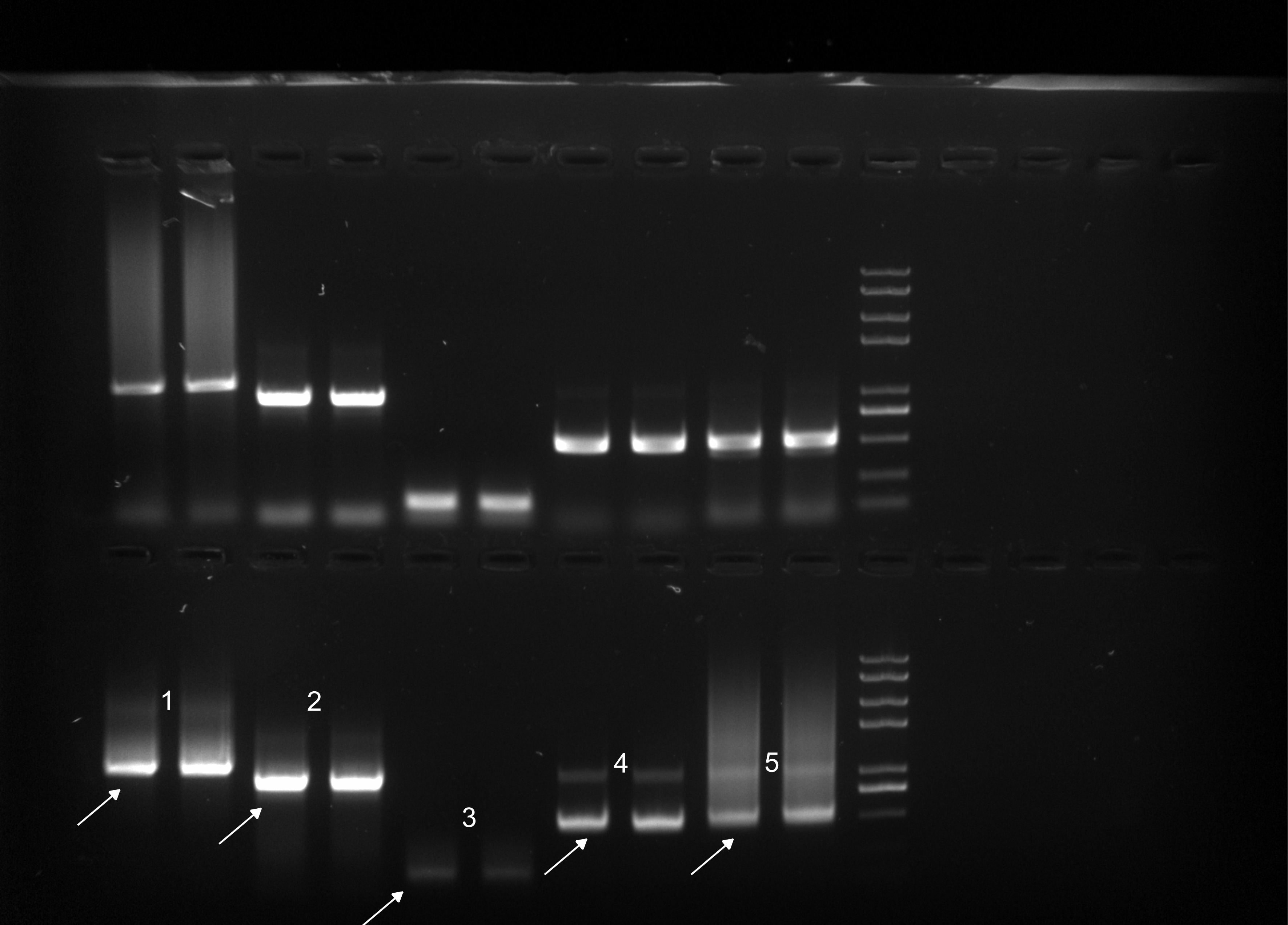

Supplement: Supplementary file 15 — Source Data [file 41467_2019_11263_MOESM15_ESM.zip › Source data-Fig. 2a.tif]

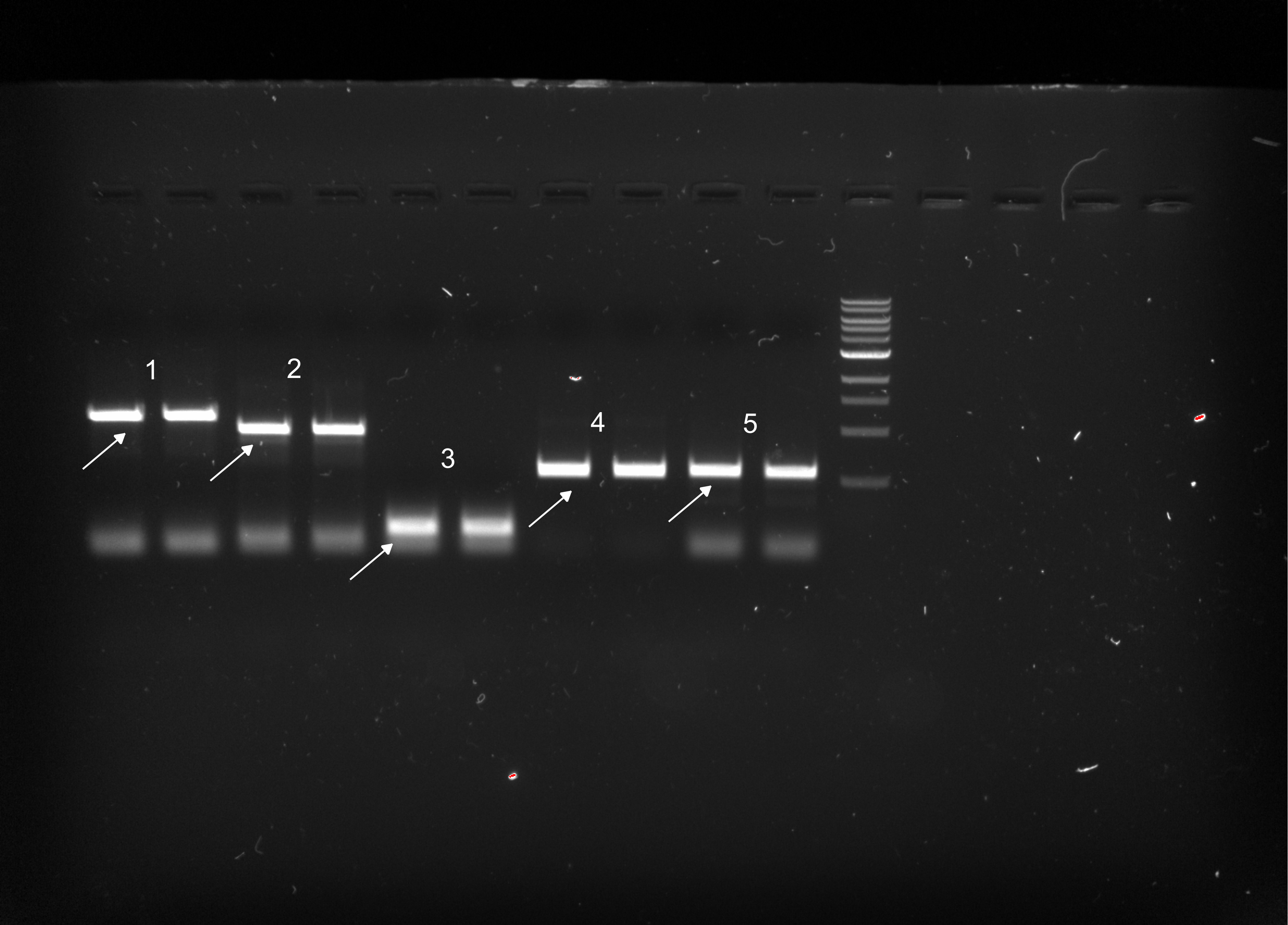

Supplement: Supplementary file 15 — Source Data [file 41467_2019_11263_MOESM15_ESM.zip › Source data-Fig. 2b.tif]

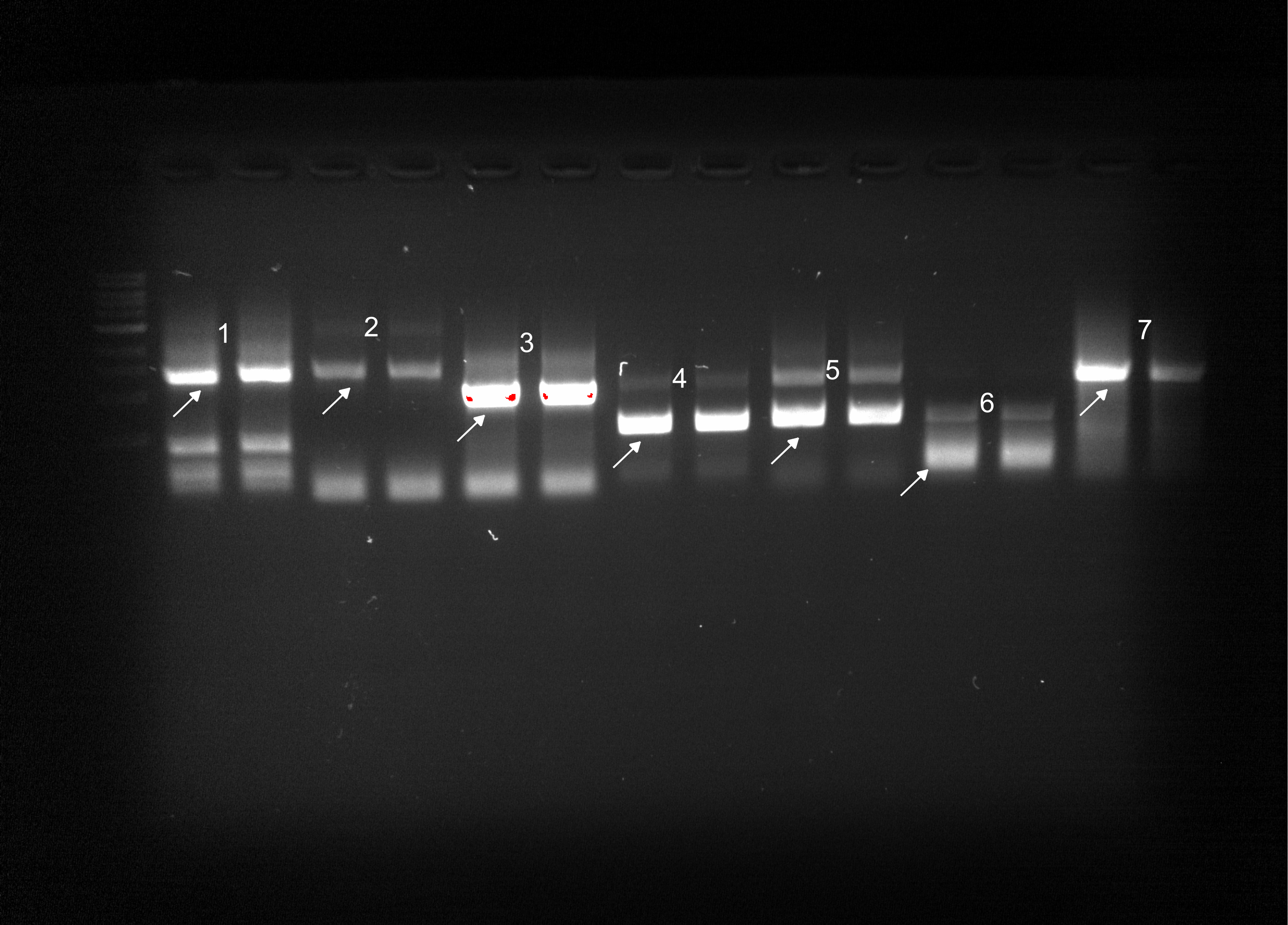

Supplement: Supplementary file 15 — Source Data [file 41467_2019_11263_MOESM15_ESM.zip › Source data-Fig. 4c.tif]
